# Genomic Characteristics and Selection Signatures in Indigenous Chongming White Goat (*Capra hircus*)

**DOI:** 10.3389/fgene.2020.00901

**Published:** 2020-08-21

**Authors:** Jun Gao, Yuhua Lyu, Defu Zhang, Kiran Kumar Reddi, Fengping Sun, Jianzhong Yi, Chengqian Liu, Hong Li, Huijuan Yao, Jianjun Dai, Fuyi Xu

**Affiliations:** ^1^Institute of Animal Husbandry and Veterinary Science, Shanghai Academy of Agricultural Sciences, Shanghai, China; ^2^Department of Genetics, Genomics, and Informatics, University of Tennessee Health Science Center, Memphis, TN, United States; ^3^Department of Bioscience Research, College of Dentistry, University of Tennessee Health Science Center, Memphis, TN, United States

**Keywords:** chongming white goat, whole-genome re-sequencing, genomic characteristics, selection signature, *Capra hircus*

## Abstract

The Chongming white goat (CM) is an indigenous goat breed exhibits unique traits that are adapted to the local environment and artificial selection. By performing whole-genome re-sequencing, we generated 14–20× coverage sequences from 10 domestic goat breeds to explore the genomic characteristics and selection signatures of the CM breed. We identified a total of 23,508,551 single-nucleotide polymorphisms (SNPs) and 2,830,800 insertion–deletion mutations (indels) after read mapping and variant calling. We further specifically identified 1.2% SNPs (271,713) and 0.9% indels (24,843) unique to the CM breed in comparison with the other nine goat breeds. Missense (SIFT < 0.05), frameshift, splice-site, start-loss, stop-loss, and stop-gain variants were identified in 183 protein-coding genes of the CM breed. Of the 183, 36 genes, including *AP4E1*, *FSHR*, *COL11A2*, and *DYSF*, are involved in phenotype ontology terms related to the nervous system, short stature, and skeletal muscle morphology. Moreover, based on genome-wide *F*_ST_ and pooled heterozygosity (*Hp*) calculation, we further identified selection signature genes between the CM and the other nine goat breeds. These genes are significantly associated with the nervous system (*C2CD3*, *DNAJB13*, *UCP2*, *ZMYND11*, *CEP126*, *SCAPER*, and *TSHR*), growth (*UCP2*, *UCP3*, *TSHR*, *FGFR1*, *ERLIN2*, and *ZNF703*), and coat color (*KITLG*, *ASIP*, *AHCY*, *RALY*, and *MC1R*). Our results suggest that the CM breed may be differentiated from other goat breeds in terms of nervous system owing to natural or artificial selection. The whole-genome analysis provides an improved understanding of genetic diversity and trait exploration for this indigenous goat breed.

## Introduction

Global livestock genetic diversity encompasses species, breeds, strains, and their variations. The diversity between and within breeds rather than species is always considered to be essential for the purposes of conservation biology (Yaro et al., 2017). Goats are important husbandry animals with an ancient domestication history and economic value. It has been reported that their domestication occurred approximately 10,000 years ago and spread worldwide following human migrations and trade routes (Benjelloun et al., 2015; Kim et al., 2019). China is the global leader in terms of goat production (Skapetas and Bampidis, 2016), including many commercial, indigenous, and composite breeds. Owing to the weaker production potential compared with that of some global commercial breeds, indigenous goat breeds are facing the problem of genetic invasion and resource degradation in recent years (Kim et al., 2019; Monau et al., 2020).

Indigenous goats are unique groups that have developed under the forces of natural selection, domestication, and local environmental conditions. Their genetic background, unique traits, and biodiversity, as well as their environmental adaptability, serve as a kind of biological heritage that can be used for the genetic improvement of animal husbandry (Kim et al., 2019). The Chongming white goat (CM) is an indigenous island-type goat breed distributed and bred on Shanghai Chongming Island. Although there have not been sufficient systematic studies on the selection signal genes for island climate adaptation, this may be a direction of research. The CM goats were also marked as special agricultural products with geographical indications registered by the State Administration for Industry and Commerce of China. Moreover, the relatively small size and ease of husbandry make it a suitable animal model for some specific human diseases such as acute spinal cord compression injuries (Huang et al., 2013). However, even with rich domestication history, studies on the genome-wide characteristics of this indigenous goat have not been reported yet to the best of our knowledge.

Whole-genome sequencing (WGS) information bypasses the study limitations of maternal mitochondrial DNA and paternal Y chromosome inheritance in species evolution and population history dynamics. Moreover, WGS can also ameliorate the current problems of breed bias and insufficient markers in genotyping chips, and the new genetic variation information also provides research materials for the further production of high-density chips. The use of WGS and reference genome alignment can identify more comprehensive, genome-wide variation, such as the presence of single-nucleotide polymorphisms (SNPs), insertion–deletion mutations (indels), and structural variants (SVs). Although high-throughput sequencing technology enables the comparison of patterns of polymorphisms at whole-genome levels, characterizing genetic structure from individual sequencing data still remains expensive for non-model species. The DNA pooling strategy (Pool-seq) represents an attractive and cost-effective alternative for SNP discovery (Futschik and Schlötterer, 2010; Hivert et al., 2018) and have been successfully applied to many domestic animal studies such as chicken (Rubin et al., 2010), pig (Rubin et al., 2012), goat (Wang et al., 2016; Guo et al., 2018), and sheep (Liu et al., 2016).

Molecular genetic markers, such as high-density SNPs, are often used to analyze genetic variation at the genome-wide level caused by natural and artificial selection of species. For example, the Chinese Tibetan cashmere goat adapted to a high-altitude area and hypoxic environment. In which, 339 genes were identified through exome sequencing, which are potentially under high-altitude selection (Song et al., 2016). Another study (Kim et al., 2019) analyzed the genome of the Korean indigenous goat (KNG) in comparison with that of nine other goat breeds and revealed that the KNG has selection signatures for *Salmonella* infection and cardiomyopathy pathways. Wang et al. (2016) generated 9–13× coverage sequences from eight domesticated goat breeds and identified 22 genomic regions that may be associated with specific phenotypic traits, including coat color, body size, cashmere traits, and high-altitude adaptation.

In this study, we aimed to explore the genomic characteristics in the CM goat. Utilizing WGS and genomic approaches, we conducted the first comparative genomic study to reveal the genome-wide variation and selection signatures in the CM breed.

## Materials and Methods

### Animals

**FIGURE 1:**
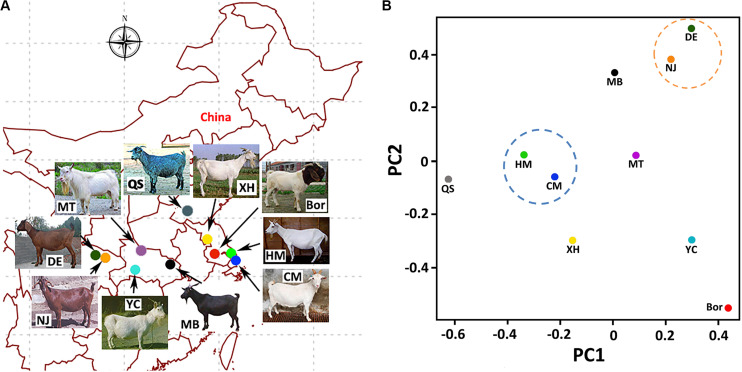
Sampling information and principal component analysis (PCA) of the 10 breeds. **(A)** Goat breeds and sampling locations. **(B)** Plot of the first two principal components (PC1 and PC2) of the 10 breeds on the basis of the detected single-nucleotide polymorphisms (SNPs).

We collected 100 goat samples from 10 breeds, with 10 individuals per breed (five bucks and five does). Samples of each breed were randomly collected from two to three local breeding farms in their province (Figure 1A). The 10 breeds included an indigenous CM breed from the farms of Shanghai Academy of Agricultural Sciences (SAAS), which were located on the Chongming Island of Shanghai. The other nine breeds were from the following regions of China: commercial Boer goats (Bor) from Jiangsu Province; Haimen goats (HM) from Jiangsu Province; Xuhuai goats (XH) from Jiangsu Province; Matou goats (MT) from Hubei Province; Da’er goats (DE) from Sichuan Province; Nanjiang yellow goats (NJ) from Sichuan Province; Macheng black goats (MB) from Hubei Province; Yichang white goats (YC) from Yichang City, Hubei Province; and Qin goats (QS) from Jining City, Shandong Province. The phenotypic descriptions of these 10 breeds can be found in Supplementary Data S1.

### Genomic DNA Extraction and Whole-Genome Sequencing

The genomic DNA was extracted from goat ear punches using the cetyl trimethylammonium bromide (CTAB) method (Thomas et al., 1997). DNA isolated from 10 animals per breed was pooled evenly (by μg of DNA) into a single sample. We ensured that the qualities of all the DNA samples were recorded as given OD260/280 = 1.8∼2.0 and OD260/230 ≧ 2.0. One microgram of pooling DNA was used for library preparation using the TruSeq^TM^ Nano DNA Library Prep Kit (Illumina, San Diego, CA, United States) according to the manufacturer’s guidelines. Briefly, the qualified DNA was randomly fragmented using ultrasound-based strategy (Larguinho et al., 2010). The fragments were then subjected to end-repair followed by adaptor ligation to the fragments. Fragments of ∼400 bp in length were selected, enriched by magnetic beads, and further amplified by polymerase chain reaction. High-throughput sequencing was performed using the Illumina HiSeq^TM^ platform (2 × 150 bp read length). The library preparation and sequencing were completed by Shanghai Majorbio Company, China^1^.

### Raw Data Quality Control and Filtering

The raw reads underwent the following filter to get the clean data: (1) remove adaptor sequences; (2) remove bases containing non-AGCT at the 5’ end; (3) trim the end of the reads with the sequencing quality score less than Q20; (4) remove reads containing *N* > 10%; and (5) discard reads with length less than 25 bp after above filtering.

After the quality control and filtering, the clean reads were summarized with the following statistics: (1) number of the reads; (2) number of the bases; (3) percentages of Q30 bases; (4) average and median insertion size; and (5) average sequencing depth.

### Genomic Alignment and Variant Calling

We used the San Clemente goat’s WGS (ARS1) (Bickhart et al., 2017) as the mapping reference genome^2^, which is the latest *de novo* assembled sequence (*Capra hircus*). The clean reads were mapped against the ARS1 by using Burrows Wheeler Alignment (BWA v0.7.15) (Li and Durbin, 2009) with default parameters. We used the Picard v2.23.1^3^ to remove duplicate reads and the GATK (v3.8.0) (McKenna et al., 2010) to call variants (SNPs and indels). The VCFtools v0.1.15 (Li et al., 2009) was used for variant filtering. More precisely, SNPs and indels were filtered by mapping quality >50 and sequencing depth ≧ 10. The BreakDancer v1.3.6 (Chen et al., 2009) was applied to detect the SVs. Variant annotation was conducted with the Ensembl Variant Effect Predictor (VEP^4^) (McLaren et al., 2016). The Sorting Intolerant from Tolerant (SIFT) was used for predicting whether an amino acid substitution affects protein function on the basis of sequence homology and the physical properties of amino acids (Sim et al., 2012).

### Gene Functional Enrichment Analysis

Gene set enrichment analysis was performed at g: Profiler^5^, a web server for functional enrichment analysis and conversions of gene lists (Raudvere et al., 2019). For gene ontology (GO; biological processes) and Kyoto Encyclopedia of Genes and Genomes (KEGG) pathway, the goat genomic annotation was used. For phenotype ontology, since this information is not available for goat, we used human phenotype ontology (HP) instead to perform the analysis. The *P*-value generated from the test was automatically adjusted to account for multiple comparisons using the Benjamini and Hochberg correction (Benjamini and Hochberg, 1995). The false discovery rate (FDR) < 0.05 was required to determine the genes that are significantly overrepresented in those categories.

### Principal Component Analysis and Detection of Selection Signature Genes

The SNP variant file was transformed to PLINK PED format via PLINK v1.9 (Purcell et al., 2007) to examine the genetic relationships with principal component analysis (PCA) among the 10 goat breeds.

We used two approaches include *F*_ST_ and pooled heterozygosity (*Hp*) to identify the selection signature differences between the genomes of the CM and the other goat breeds. The weighted population pairwise *F*_ST_ values were calculated for each SNP according to the formula of $F_{\text{ST}}=\frac{S^{2}}{\bar{p}\,\left(1-\bar{p}\right)+S^{2}/r}$, in which *S*^2^ represents the sampling variance of allele frequencies between two breeds and $\bar{p}$ represents the overall average allele frequency across breeds (Weir and Cockerham, 1984; Guo et al., 2018). *F*_ST_ values were further averaged over SNPs using a 100-kb sliding window with a 50-kb step size and *Z*-transformed as follows: *ZF*_ST_ = (*F*_ST_−μ*F*_ST_)/σ*F*_ST_, where μ*F*_ST_ and *σF*_ST_ are the mean and standard deviation for the *F*_ST_, respectively. This analysis was done with popoolation2 v1.201 (Kofler et al., 2011) and R package v3.5.1 (R Core Team, 2013).

The identification of selective sweeps was performed by calculating the *ZHp* for each pool and SNP. The numbers of reads corresponding to the major (*n*_M__aj_) and minor (*n*_*Min*_) allele frequencies and for each window in each breed pool, the *Hp* score is calculated according to the formula of *Hp* = 2 ∑*n*_Maj_∑*n*_Min_/(∑*n*_Maj_ + ∑*n*_Min_)^2^, where ∑*n*_Maj_, and ∑*n*_Min_ are the sums of *n*_*Maj*_ and *n*_*Min*_ for all SNPs in the window. Individual *Hp* values were *Z* transformed as follows: *ZHp* = (*Hp* - μ*Hp*)/*σHp*, where μ*Hp* and *σHp* are the mean and standard deviation for the *Hp*, respectively. Consistent with the *F*_ST_, we calculated the *ZHp* value using a 100-kb sliding window with a 50-kb step size.

Windows with extremely high *F*_ST_ values (99% percentile) and low *ZHp* scores (1% percentile) were proposed to be under selection. The genome-wide scan results of *ZF*_ST_ and *ZHp* can be found in Supplementary Data S2, S3.

## Results

### Read Mapping and Variant Identification

In this study, genome sequencing yielded a total of 652.25 Gb of raw data from the 10 goat breeds. Approximately 195.6 (Bor) to 253 (MT) million clean reads were obtained after quality control and filtration. More than 97.5% clean reads were mapped against to the reference genome ARS1 with 5× coverage of ∼94%. The average sequencing depth ranges from 14.24 × (YC) to 20.3 × (QS). These results indicated that high-quality sequences were obtained (Table 1).

**TABLE 1:** Summary statistics for sequencing results.

Sample ID	Clean reads	GC (%)	Q30 (%)	Properly mapped (%)	Duplicate ratio (%)	Coverage (5×),%	Average depth
CM	196,441,962	43.51	92.9	98	29.05	93.11	14.31
HM	200,151,772	43.49	92.61	97.97	27.31	92.96	14.94
XH	208,253,056	43.55	92.87	98.25	29.57	93.78	15.07
YC	189,344,096	43.73	92.45	97.94	26.57	93.23	14.24
MT	253,043,449	42.64	94.12	97.61	26.95	94.17	19.02
QS	250,986,198	43.82	93.41	98.21	21.24	94.17	20.3
MB	231,897,959	42.57	94.26	97.59	25.65	93.91	17.75
DE	244,000,307	43.58	93.47	97.55	24.81	94.06	18.8
NJ	199,979,440	43.56	92.85	97.97	25.14	93.49	15.35
Bor	195,608,801	43.51	92.46	98.01	24.85	93.4	15.06

*GC, guanine–cytosine.*

A total of 23,508,551 SNPs and 2,830,800 indels were detected, out of which 12,939,052 SNPs and 1,571,878 indels were identified in the CM breed. The number of SNPs in the other nine breeds ranged from 11,871,653 (Bor) to 13,597,084 (MT). Similarly, the lowest number (1,486,903) of indels was detected in the Bor breed and the largest number (1,704,999) in the MT breed. By comparing the other nine breeds, we further identified 1.2% SNPs (271,713) and 0.9% indels (24,843) unique to the CM breed. The other nine breeds’ specific variants ranged from 259,098 (MB) to 540,055 (QS) for SNPs, and 26,031 (NJ) to 59,999 (QS) for indels (Table 2).

**TABLE 2:** Statistics of the variants identified in the 10 goat breeds.

Sample ID	SNP	Breed-specific SNP	Indel	Breed-specific Indel	SV	
					Deletion	Insertion
CM	12,939,052	271,713	1,571,878	24,843	256	0
HM	12,666,186	319,560	1,560,507	30,478	250	0
XH	13,323,084	486,342	1,608,184	42,103	378	2
YC	12,715,233	278,306	1,555,794	26,130	221	15
MT	13,597,084	306,317	1,704,999	32,932	831	10
QS	12,310,457	540,055	1,589,967	59,999	446	0
MB	13,104,219	259,098	1,654,422	28,732	635	7
DE	13,543,087	429,052	1,683,457	41,843	638	18
NJ	13,093,329	274,265	1,602,744	26,031	310	2
Bor	11,871,653	364,314	1,486,903	36,335	417	6

*SNP, single-nucleotide polymorphism; SV, structural variant.*

Based on the PCA results (Figure 1B), the CM breed showed the closest relationship with the HM breed. The Bor breed demonstrated the most divergent genetic relationships with the CM breed. This is consistent with the fact that the Bor goat is originated from South African regions and was introduced domestically as an excellent commercial meat goat (Malan, 2000). The DE and NJ breeds are found in the Sichuan Province of China and are clustered together on the PCA map. This was also in line with our expectations.

**FIGURE 2:**
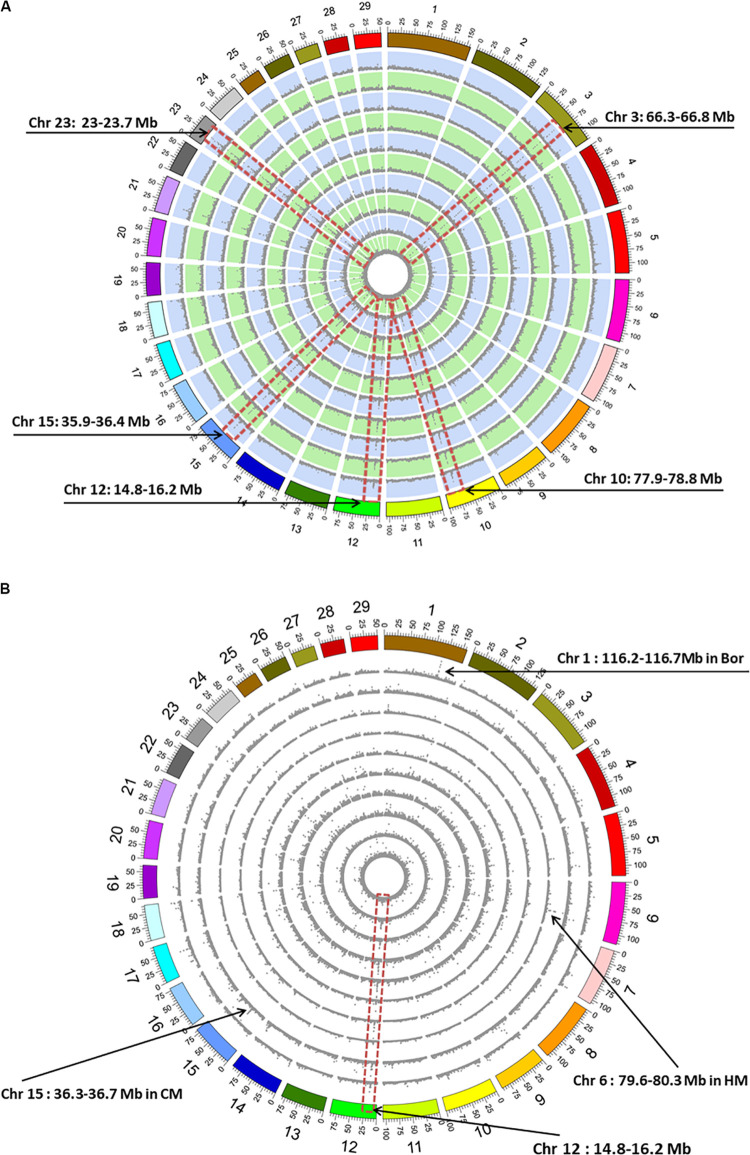
Genome-wide distribution histograms of single-nucleotide polymorphism (SNP) density counted with 100-kb non-overlapping windows. **(A)** SNP density. **(B)** Breed-specific SNP density. The outermost circle represents the reference genome (ARS1). The circles from outermost to innermost show the genome-wide distribution of SNP density for the Bor, CM, HM, XH, QS, MB, MT, NJ, DE, and YC breeds. The red dotted frame indicates the consistent SNP high-density location on a specific chromosome (Chr). Mb, megabase; Bor, commercial Boer goats; CM, Chongming white goat; HM, Haimen goats; XH, Xuhuai goats; QS, Qin goats; MB, Macheng black goats; MT, Matou goats; NJ, Nanjiang yellow goats; DE, Da’er goats; YC, Yichang white goats.

We calculated genome-wide SNP distributions, counted in 100-kb non-overlapping windows, to explore the highly polymorphic regions for the goat breeds. The results demonstrated the average SNP density was ∼5 SNPs/kb among all the 10 breeds. We identified five common high-polymorphic regions with average SNP density >15 SNPs/kb, including 66.3–66.8 Mb on chromosome (Chr) 3, 77.9–78.8 Mb on Chr 10, 14.8–16.2 Mb on Chr 12, 35.9–36.4 Mb on Chr 15, and 23–23.7 Mb on Chr 23 (Figure 2A). For the breed-specific SNPs, the average density was approximately 0.13 SNPs/kb, and only Chr 12: 14.8–16.2 Mb region shows similar high-polymorphic characteristics among the 10 breeds (Figure 1B). Moreover, several breed-specific, highly polymorphic regions were identified, such as 116.2–116.7 Mb on Chr 1 (average density >2.6 SNPs/kb) in Bor, 36.3–36.7 Mb on Chr 15 in CM (average density >1.4 SNPs/kb), and 79.6–80.3 Mb on Chr 6 (average density >3.5 SNPs/kb) in HM (Figure 2B).

### Variant Annotation

**FIGURE 3:**
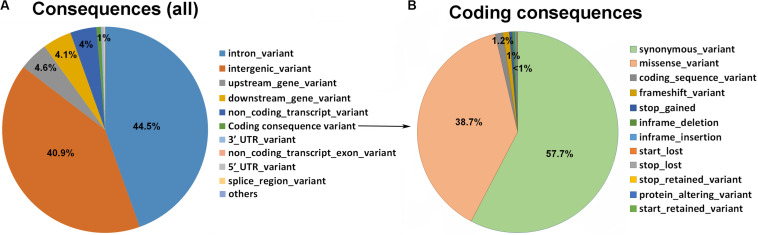
Statistics regarding the variant types of the goat whole genome using VEP Ensemble gene annotation. **(A)** Pie plot of the total variant annotation. **(B)** Pie plot of the coding consequence variant annotation.

We performed the variant annotation using VEP, which indicated that 40.9% of variants (SNPs and indels) were located in intergenic regions and the rest in genic regions, including introns (44.5%), upstream (4.6%), and downstream (4.1%) (Figure 3A). Of the total variants identified, ∼0.7% were coding consequence variants (271,960). Of these, 57.7% were synonymous variants (156,873); 38.7% were missense variants (105,257); 1.2% were coding sequence variants (3,371); 1% were frameshift variants (2,816); and ∼0.45% were stop-gained variants (1,243) (Figure 3B). Among the missense variants, ∼15,000 predicted having deleterious effects on protein structure [Sorting Intolerant from Tolerant (SIFT) < 0.05].

**FIGURE 4:**
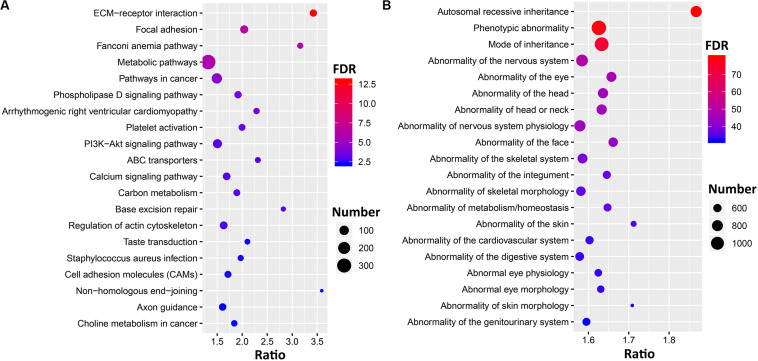
Gene set enrichment analysis for the genes with loss-of-function variants. Bubble plots showing the top 20 significant results [false discovery rate (FDR) < 0.05] for Kyoto Encyclopedia of Genes and Genomes (KEGG) pathways **(A)** and human phenotype ontology (HP) terms **(B)**. The circles represent the number of enriched genes, and the colors represent log10 transformed FDR values.

Filtering resulted in 4,703 genes that harbor ∼20,000 loss-of-function (LoF) variants, including missense (SIFT) < 0.05, frameshift, start-loss, stop-gain or stop-loss, and splice-site variants (Supplementary Data S4). To further understand the function and pathways, we submitted those genes to g: Profiler and performed gene set enrichment analysis. Those genes significantly enriched in the GO terms of biological process (FDR = 9.85E-78), cellular process (FDR = 4.68E-29), and biological regulation (FDR = 7.75E-23). The KEGG pathways (Figure 4A) highlighted the genes significantly enriched in the extracellular matrix (ECM)–receptor interactions (FDR = 1.12E-13), focal adhesion (FDR = 4.47E-7), and metabolic pathways (FDR = 1.69E-6). Phenotype ontology (Figure 4B) suggests these genes are involved in nervous system physiology (FDR = 9.161E-50), skeletal morphology (FDR = 5.39E-37), and the cardiovascular system (FDR = 6.80E-34).

### Candidate Coding Functional Genes and Related Phenotypes in the Chongming White Goat Breed

**FIGURE 5:**
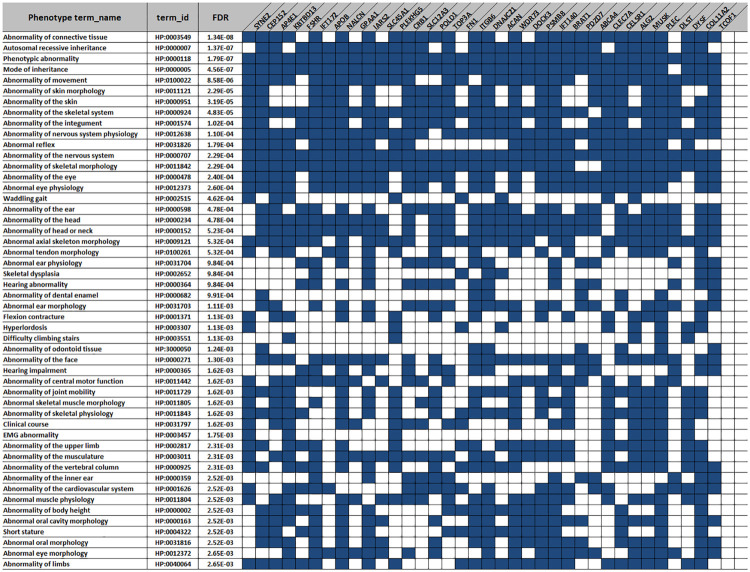
The top 50 phenotype terms for the 36 Chongming white goat (CM) breed-specific genes with the loss-of-function (LoF) variants. Blue squares indicate the genes (columns) involved in the specific phenotype terms (rows).

The CM breed is our research of interest. Thus, we aimed to further investigate the phenotypes related to the CM-specific genes with LoF variants. In total, 351 LoF variants in 183 coding genes were identified (Supplementary Data S5). We performed the phenotype enrichment analysis for those genes on g: Profiler and obtained 338 HP terms with FDR < 0.05. Next, we filtered the genes with those involved in more than 50 HP terms. This resulted in 36 out of 183 genes. The top 50 phenotype terms related to those 36 genes are listed in Figure 5. Of note, all the genes participated in regulation of the nervous system (HP:0000707, FDR = 0.0002). Furthermore, most of the genes were associated with several other phenotype terms. For instance, 20 genes (e.g., *SYNE2*, *AP4E1*, and *KBTBD13*) were enriched in the “skeletal muscle morphology” (HP:0011805, FDR = 0.0016); 18 genes (e.g., *AP4E1*, and *FSHR*, and *Col11a2*) involved in the “short stature” (HP:0004322, FDR = 0.0025) and “body height” (HP:000002, FDR = 0.0025); and 15 (e.g., *IFT172*, *NALCN*, and *IARS2*) genes are found in the terms of “ear morphology” (HP:0031703, FDR = 0.001106) and “hearing” (HP:0000364, FDR = 0.00098), simultaneously.

### Selection Signature Genes

#### Co-selection Signature Genes

**FIGURE 6:**
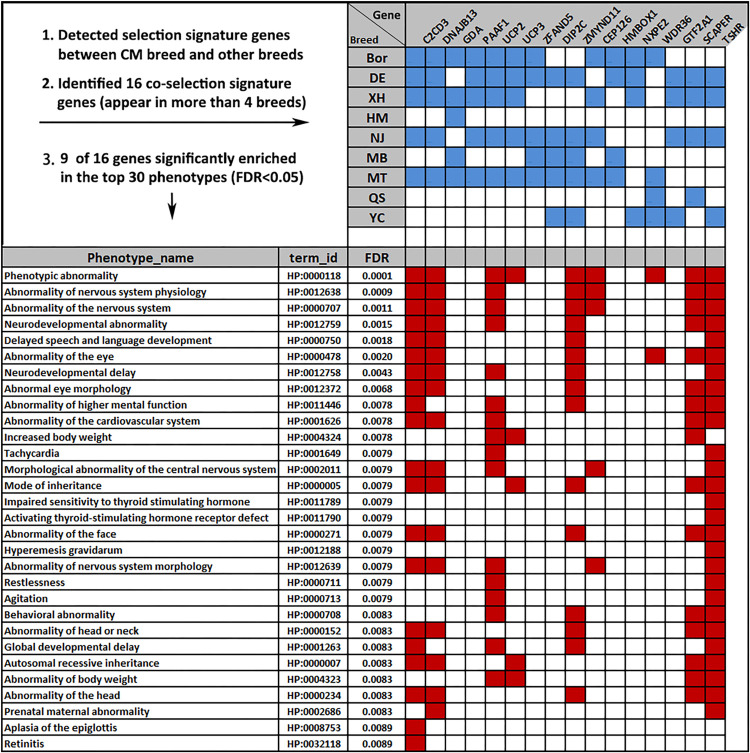
Phenotype overview of the 16 co-selection signature genes. Blue square indicates genes appearing in more than four of 10 breeds [false discovery rate (FDR) < 0.05]. Red squares indicate the nine genes enriched in the top 30 phenotype terms.

The CM goats are domesticated under the natural conditions of Chongming Island in Shanghai. In order to detect the genome-wide selection signatures, we calculated *F*_ST_ values between the CM and each of the other nine breeds using a 100-kb sliding widow with a step size of 50 kb. The 99% percentile *Z* score transformed *F*_ST_ values for each comparison was calculated and averaged (*ZF*_ST_ = 3.27). This value was used as the selection signature cutoff threshold. On the basis of this, we identified hundreds of genes that underwent selection, ranging from 228 between CM and YC to 330 between CM and DE. Of those, 37 genes were observed having co-selection signatures in more than four comparisons. In addition to *F*_ST_, we also calculated the *ZHp* scores for each breed and identified 16 out of the 37 co-selection genes with low *ZHp* scores < −3 (1% percentile, Figure 6). Of those, seven genes (*C2CD3*, *DNAJB13*, *UCP2*, *ZMYND11*, *CEP126*, *SCAPER*, and *TSHR*) are related to six nervous system-related terms (HP:0012758, HP:0012759, HP:0000707, HP:0012639, HP:0012638, and HP:0002011). Four genes (*UCP2*, *UCP3*, *SCAPER*, and *TSHR*) are related to the body weight (HP:0004324 and HP:0004323), and four genes (*C2CD3*, *UCP2*, *ZMYND11*, and *TSHR*) are involved in modulating the phenotype of globe developmental delay (HP:0001263).

**FIGURE 7:**
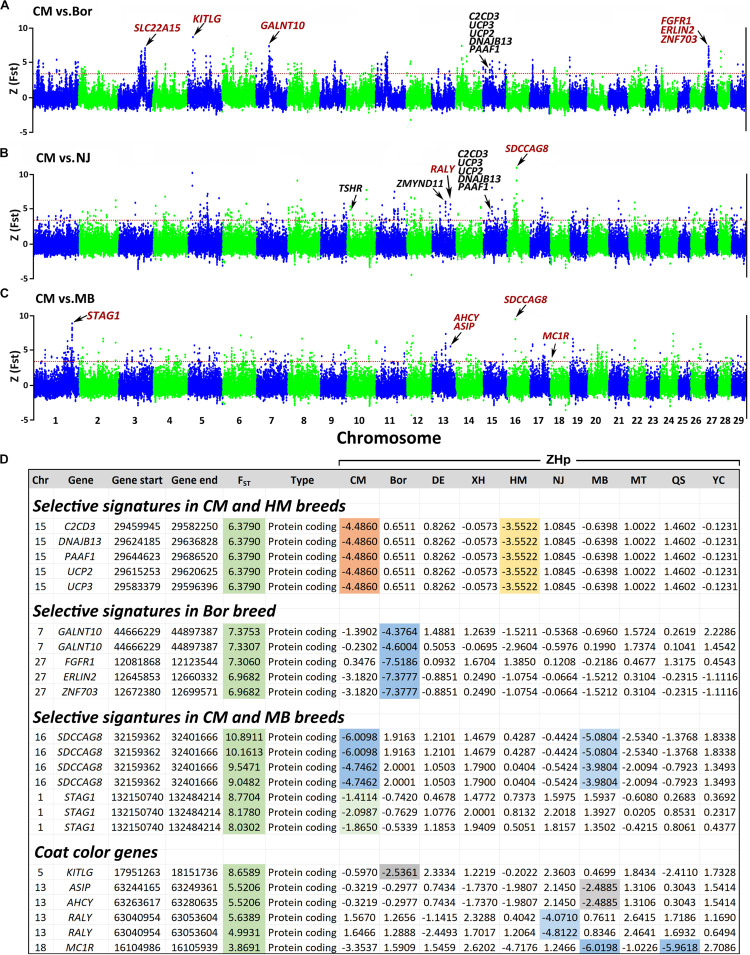
Genome-wide distributions of selection signatures in the CM vs Bor **(A)**, CM vs NJ **(B)**, and CM vs MB **(C)**. *ZHp* scores of selection genes across the breeds **(D)**. The *ZF*_ST_ and *ZHp* calculated for each sliding 100-kb window with steps of 50 kb across all autosomes. The red horizontal dashed line indicates the *ZF*_ST_ threshold value of 3.27. CM, Chongming white goat; Bor, commercial Boer goats; NJ, Nanjiang yellow goats; MB, Macheng black goats.

Notably, both *F*_ST_ and *ZHp* indicate that the 29.46–29.65 Mb region on Chr 15 has a strong selection signature in both the CM and HM breeds (Figure 7). This region contains five genes, with *C2CD3*, *DNAJB13*, and *UCP2* involved in six phenotypes of the nervous system including abnormality of nervous system physiology (HP:0012638), abnormality of nervous system (HP:0000707), neurodevelopmental abnormality (HP:0012759), neurodevelopmental delay (HP:0012758), morphological abnormality of the central nervous system (HP:0002011), and abnormality of nervous system morphology (HP:0012639).

#### Unique Selection Signature Genes in Chongming White Goat Breed

Through pairwise *F*_ST_ comparisons between the CM and the other breeds, we also identified several unique selection genes such as *SLC22A15*, *KITLG*, *GALNT10*, *FGFR1*, *ERLIN2*, and *ZNF703* in CM vs Bor (Figure 7A). Among the notable ones are *FGFR1*, *ERLIN2*, and *ZNF703* located on Chr 27 at 12–12.7 Mb, which is a unique strong selection signal with high *F*_ST_ value (>7) and low *ZHp* scores (<-7.4) only observed in the Bor breed. Moreover, *SDCCAG8* and *STAG1* with high *ZF*_ST_ values and low *ZHp* scores were detected in the CM and MB breeds. Five coat color-related genes (*KITLG*, *ASIP*, *AHCY*, *RALY*, and *MC1R*) were identified in the Bor, NJ, and MB breeds (Figure 7). The *KITLG* gene located on Chr 5 at 17.95–18.15 Mb showed a unique selective signal with *F*_ST_ value of 8.66 and *ZHp* score of −2.54 in Bor. *ASIP*, *AHCY*, and *RALY* on Chr 13 at 63–63.25 Mb demonstrated with similar *F*_ST_ values of ∼5.5. The −2.49 *ZHp* score of *ASIP* and *AHCY* in the MB breed and −4.81 *ZHp* score of *RALY* in the NJ breed were detected. In addition, *MC1R* with *F*_ST_ = 3.87 and *ZHp* = −6 in the MB and QS breeds were identified.

## Discussion

### Genetic and Genomic Studies of the Goat

In this study, we used high-throughput sequencing technologies to perform WGS for 10 goat breeds, pooling libraries from 100 goat individuals. After genome-wide mapping and variant calling, we obtained ∼23.5 million SNPs and 2.8 million indels from the 10 goat breeds. By calculating the distribution of genome-wide SNP polymorphisms in the 100-kb non-overlapping window, we identified five common chromosomal regions with high polymorphism on goat genome. The results are consistent with those reported in previous goat genome study (Guo et al., 2018). Moreover, several breed-specific, highly polymorphic regions were identified. Most of the annotated genes in these genomic regions are uncharacterized LOC symbols, and the biological importance of them remains to be elucidated.

### Genomic Characteristics of the Chongming White Goat Breed

Many indigenous breeds, which might represent highly valuable biodiversity resources, are threatened with extinction owing to their substitution by cosmopolitan breeds. The CM goat also faces this dilemma. In our study, we identified 271,713 CM-specific SNPs and 24,843 indels, which is the first whole-genome study to the best of our knowledge to characterize genetic polymorphisms in this breed. The results clearly demonstrate that the CM breed has the closest phylogenetic relationship with the HM and the most divergent relationships with the Bor breed. It has also been argued that both the CM and HM breeds belong to the Yangtze River Delta white goat species in China. The phylogenetic results among these breeds are also consistent with the expectation from the geographical distribution.

LoF variants are typically thought to have deleterious effects on protein-coding genes. The effects of these variants upon protein structure and expression in turn lead to phenotypic differences. In this study, we identified 183 genes with the CM breed-specific LoF variants. Out of these 183, 36 genes are enriched in more than 50 phenotype terms, including “nervous system,” “short stature,” and “skeletal muscle morphology” (Figure 5). The genes significantly enriched in the “nervous system” phenotypes, possibly reflecting the fact that the most critical phenotypic changes during the initial steps of animal domestication affect brain and neuronal development. Variants in these genes may produce behavioral traits that ease the domestication process or be a product of domestication itself (Carneiro et al., 2014).

The body size of the CM breed is relatively small (mature weights for bucks and does are 23–32 and 18–25 kg, respectively) compared with the Bor breed (mature weights for bucks and does are 90–130 and 80–100 kg, respectively (Lu, 2001). It is worth noting that 18 genes (e.g., *AP4E1*, *FSHR*, and *COL11A2*) are related to the terms of “short stature” and “body height.” Mutations in the *AP4E1* gene are associated with neurodevelopmental disorders in humans, which are characterized by intellectual disability, global developmental delay, and short stature (Jamra et al., 2011; Moreno-De-Luca et al., 2011). Moreover, *Ap4e1* mutant mice display a lean body mass phenotype (Eppig et al., 2005). Follicle-stimulating hormone receptor (FSHR) plays a prominent role in mammalian reproduction (Papadimitriou et al., 2016), and ovarian follicle *FSHR* expression levels in prolific Lezhi black goats were reported to be significantly higher than those in non-prolific Tibetan goats (Zi et al., 2013). In mice, *Fshr* mutant females have body weights 20% greater than those of wild-type control females (Danilovich et al., 2000). Similarly, homozygote *Col11a2* mutant mice are 25% smaller at birth than their wild-type counterparts (Eppig et al., 2005).

In addition, skeletal muscle is a significant contributor to overall body mass and an important structural component of the mammalian body (Berridge et al., 2018). Dysferlin (encoded by *DYSF*) is a skeletal muscle protein important for organization and structure of the sarcolemma (Spuler et al., 2008). *DYSF* mutations are associated with muscular dystrophy in humans (Bashir et al., 1998) and mice (Bittner et al., 1999). *DYSF* has also been identified as an alternatively spliced gene in goat leg muscle, likely playing crucial roles in skeletal muscle development (Xu et al., 2018). A study has revealed several candidate genes associated with goat ear morphology (Brito et al., 2017). In our study, 15 genes were related to the terms of “ear morphology” and “hearing” simultaneously. Therefore, these 36 genes may play important roles in the CM breed-specific phenotypic traits, and study of the biological underpinnings of these genes in relation to the traits is necessary and important.

### Selection Signature Genes of the Chongming White Goat Breed

#### Co-selection Signature Genes

Through calculated *F*_ST_ values, we filtered 37 genes that have co-selection signals, 16 of which had *ZHp* scores < -3, which indicate that these genes have significant selection signatures with a high degree of confidence. Moreover, seven genes (*C2CD3*, *DNAJB13*, *UCP2*, *ZMYND11*, *CEP126*, *SCAPER*, and *TSHR*) were significantly enriched in six nervous system-related phenotypes (Figure 6). Some of them were reported to have clear functions in human or mouse studies. For example, *ZMYND11* variants were identified in mice with anxiety-like behavior (Parker et al., 2016). Pathogenic variants in *ZMYND11* have been associated with intellectual disability, behavioral abnormalities, and seizures in humans (Yates et al., 2020).

The region on Chr 15 at 29.46–29.65 Mb contains five genes (*C2CD3*, *DNAJB13*, *UCP2*, *UCP3*, and *PAAF1*), which is a strong selective signal region only in the CM and HM breeds. Given their close phylogenetic relationship, and the fact that three genes (*C2CD3*, *DNAJB13*, and *UCP2*) are implicated in six nervous system-related traits, we have reason to suppose that the nervous system in the CM and HM breeds may differ from that in the other goat breeds. Owing to the lack of sufficient phenotypic information on the nervous system of goats, the contribution of these genes is still unclear and remains to be further elucidated.

As mentioned above, it is of interest to identify genes important for goat growth and development and how these genes differ between breeds. Five selection signature genes (*UCP2*, *UCP3*, *ZMYND11*, *SCAPER*, and *TSHR*) are contributing to the phenotype term of “body weight” or “global developmental delay.” Our results suggested that thyroid-stimulating hormone receptor (TSHR) is one of the co-selective signature genes between the CM and the DE, XH, NJ, and YC breeds (Figure 6). *TSHR* has a pivotal role in metabolic regulation and photoperiod control of reproduction in vertebrates. Studies had identified *TSHR* under selective pressure in other domestic animals, such as chicken (Rubin et al., 2010) and sheep (Liu et al., 2016). Accumulating evidence suggests that the uncoupling proteins *UCP2* and *UCP3* have a role in thermogenesis and energy efficiency (Nagy et al., 2004; de Almeida Brondani et al., 2014). Thus, these five genes, which we identified in this study, may play vital roles in the growth, performance, and metabolism of the goat, but the molecular mechanisms have not been fully elucidated yet.

#### Unique Selection Signature Genes in Chongming White Goat Breed

Some selection signatures were only identified between one and two breeds. There may be a connection between these unique selective signature genes and the traits of a specific breed. For example, Bor goats are excellent for meat production, grow quickly, and have high fertility rates. We detected a unique selective region located on Chr 27 at 12–12.7 Mb, which includes three genes (*FGFR1*, *ERLIN2*, and *ZNF703*) (Figures 7A,D). Genetic studies have placed the *FGFR1* gene at the top of major ontogenic pathways that enable gastrulation, tissue development, and organogenesis (Terranova et al., 2015). Moreover, the genomic region that includes *ERLIN2* and *ZNF703* was reported to be associated with average daily gain (ADG) in Nellore cattle (Olivieri et al., 2016). Although the function of these unique genes and the relationship with the characteristics of the goat breeds are still unclear, these unique strongly selective genes that include *SDCCAG8* and *STAG1* in the CM and MB breeds are worthy of further study.

#### Coat Color Genes

Animal coat color is an important trait that is easy to observe. Studies have shown that some coat color genes have been underwent selections. We identified five coat color-related genes that have selection signatures in this study. The *KITLG* gene shows a strong selection signature in the Bor breed with unique high *F*_ST_ and low *ZHp* scores (Figures 7A,D). *KITLG* encodes for the ligand of c-Kit, which is associated with coat color in pigs (Hadjiconstantouras et al., 2008) and UV-protective eye area pigmentation in cattle (Pausch et al., 2012). *KITLG* genomic region also reported has a significant effect on skin and hair color of humans (Miller et al., 2007; Sulem et al., 2007). *RALY* was reported to be associated with saddle tan and black-and-tan phenotypes in the dogs (Dreger et al., 2013). The *RALY-*ELF2S2**-*ASIP* locus has influence on skin and hair pigmentation in the Nanjiang yellow goat (Guo et al., 2018). In the present study, we also detected the selective signal of *RALY* in the NJ breed, which further emphasizes that the *RALY* gene may contribute to the tan color of goats. Agouti signaling protein gene (*ASIP*) result in various coat patterns in domestic mammals^6^ including mouse (Voisey and Van Daal, 2002), dog (Kerns et al., 2004), cat (Eizirik et al., 2003), rabbit (Fontanesi et al., 2010), horse (Rieder et al., 2001), sheep (Norris and Whan, 2008), and donkeys (Abitbol et al., 2015). It was also studied as a strong candidate gene associated with the black coat color in a Chinese Taihang black goat (Wang et al., 2016). In this study, only the MB breed has an all-black coat, and the results suggest that the *ASIP* and *AHCY* genes may contribute to the black coat color of the MB breed. For the *MC1R* gene, we observed the low *ZHp* values in the MB (black) and QS breeds (blue gray). The melanocortin one receptor (MC1R) is a melanocytic Gs protein-coupled receptor that regulates skin pigmentation, UV responses, and melanoma risk (Wolf Horrell et al., 2016). Studies have shown that this gene is important for regulation of eumelanin (black/brown) and phaeomelanin (red/yellow) syntheses in mammalian melanocytes (Wang et al., 2016). The coat color of animals may be determined by the combined action of several genes. These five genes are present in varying degrees of selective signatures in our 10 goat breeds, which highlighted that they play important roles in determining the coat color phenotypes of goats.

## Conclusion

In this study, by using the WGS method and genomic approaches, we conducted a comparative genomic study for the first time to reveal the genomic characteristics and selection signatures between the CM and the other nine goat breeds. Our results identified 36 genes with the CM breed-specific LoF variants that were significantly enriched in the phenotype terms of “nervous system,” “short stature,” and “skeletal muscle morphology.” From another perspective, hundreds of selection signature genes were identified in this study that are possibly associated with important traits, such as growth (*UCP2*, *UCP3*, *ZMYND11*, *SCAPER*, *TSHR*, *FGFR1*, *ERLIN2*, and *ZNF703*), nervous system (*C2CD3*, *DNAJB13*, *UCP2*, *ZMYND11*, *CEP126*, *SCAPER*, and *TSHR*), and coat color (*KITLG*, *ASIP*, *AHCY*, *RALY*, and *M1CR*). The genes with functional mutations and the selection signatures explored in this study both reflect the fact that the nervous system of the CM breed may differ from the other breeds. Although the trait-related genes require further validation through biological experiments, our findings provide an improved understanding for biodiversity conservation and trait exploration for the domestic goats.

## Data Availability Statement

Raw sequence data of 10 sheep breeds in the bam format from this study were deposited in the NCBI Short Read Archive (SRA; http://www.ncbi.nlm.nih.gov/sra) under accession number PRJNA608539.

## Ethics Statement

The animal study was reviewed and approved by Experimental Animal Management Committee of SAAS.

## Author Contributions

JG and JD managed the project. FX and JG carried out the bioinformatics analyses. YL, DZ, FS, JY, CL, HL, and HY collected the samples. JG and FX wrote the manuscript. KR reviewed and edited the English writing. All authors read and approved the final manuscript.

## Conflict of Interest

The authors declare that the research was conducted in the absence of any commercial or financial relationships that could be construed as a potential conflict of interest.

## Footnotes

1 www.majorbio.com

2 www.ncbi.nlm.nih.gov/assembly/GCF_001704415.1

3 https://broadinstitute.github.io/picard

4 https://useast.ensembl.org/Tools/VEP

5 https://biit.cs.ut.ee/gprofiler/gost

6 http://omia.angis.org.au

## Supplementary Material

DATA S1 Phenotype description of 10 goat breeds.

DATA S2 *ZF*_ST_ and *Z*_Hp_ results.

DATA S3 *ZF*_ST_ and *Z*_Hp_ results.

DATA S4 4703 genes harbor LoF variants.

DATA S5 183 CM specific genes with LoF variants.
